# Expanding Telemonitoring in a Virtual World: A Case Study of the Expansion of a Heart Failure Telemonitoring Program During the COVID-19 Pandemic

**DOI:** 10.2196/26165

**Published:** 2021-01-22

**Authors:** Sahr Wali, Milena Guessi Margarido, Amika Shah, Patrick Ware, Michael McDonald, Mary O'Sullivan, Juan Duero Posada, Heather Ross, Emily Seto

**Affiliations:** 1 Centre for Global eHealth Innovation Techna Institute University Health Network Toronto, ON Canada; 2 Institute of Health Policy, Management and Evaluation Dalla Lana School of Public Health University of Toronto Toronto, ON Canada; 3 Department of Computer Systems Institute of Mathematics and Computer Science University of São Paulo São Paulo Brazil; 4 Peter Munk Cardiac Centre University Health Network Toronto, ON Canada; 5 Ted Rogers Centre for Heart Research University Health Network Toronto, ON Canada; 6 Advanced Heart Failure, Transplantation and Mechanical Circulatory Support Toronto Western Hospital University Health Network Toronto, ON Canada; 7 Department of Medicine University of Toronto Toronto, ON Canada

**Keywords:** telemedicine, telehealth, digital health, digital medicine, virtual care, COVID-19, coronavirus, SARS-CoV-2, public health, surveillance, outbreak, pandemic, infectious disease, cardiology, patient

## Abstract

**Background:**

To minimize the spread and risk of a COVID-19 outbreak, societal norms have been challenged with respect to how essential services are delivered. With pressures to reduce the number of in-person ambulatory visits, innovative models of telemonitoring have been used during the pandemic as a necessary alternative to support access to care for patients with chronic conditions. The pandemic has led health care organizations to consider the adoption of telemonitoring interventions for the first time, while others have seen existing programs rapidly expand.

**Objective:**

At the Toronto General Hospital in Ontario, Canada, the rapid expansion of a telemonitoring program began on March 9, 2020, in response to COVID-19. The objective of this study was to understand the experiences related to the expanded role of a telemonitoring program under the changing conditions of the pandemic.

**Methods:**

A single-case qualitative study was conducted with 3 embedded units of analysis. Semistructured interviews probed the experiences of patients, clinicians, and program staff from the Medly telemonitoring program at a heart function clinic in Toronto, Canada. Data were analyzed using inductive thematic analysis as well as Eakin and Gladstone’s value-adding approach to enhance the analytic interpretation of the study findings.

**Results:**

A total of 29 participants were interviewed, including patients (n=16), clinicians (n=9), and operational staff (n=4). Four themes were identified: (1) providing care continuity through telemonitoring; (2) adapting telemonitoring operations for a more virtual health care system; (3) confronting virtual workflow challenges; and (4) fostering a meaningful patient-provider relationship. Beyond supporting virtual visits, the program’s ability to provide a more comprehensive picture of the patient’s health was valued. However, issues relating to the lack of system integration and alert-driven interactions jeopardized the perceived sustainability of the program.

**Conclusions:**

With the reduction of in-person visits during the pandemic, virtual services such as telemonitoring have demonstrated significant value. Based on our study findings, we offer recommendations to proactively adapt and scale telemonitoring programs under the changing conditions of an increasingly virtual health care system. These include revisiting the scope and expectations of telemedicine interventions, streamlining virtual patient onboarding processes, and personalizing the collection of patient information to build a stronger virtual relationship and a more holistic assessment of patient well-being.

## Introduction

### Shifting Societal Views on Essential Health Services

The unprecedented magnitude of the COVID-19 pandemic has shifted the focus of the health care system toward patients infected with SARS-CoV-2 [1]. Through these uncertain times, to minimize the spread and risk of a COVID-19 outbreak, societal norms have been challenged in terms of what health services are considered *essential* [2]*.* Specifically, health care systems have been faced with a significant challenge whereby in-person visits, surgeries, and treatments once considered *essential* have either been cancelled or postponed to reduce the likelihood of virus transmission [2,3]. With the implementation of these public health measures, patients with chronic conditions relying on traditional models of clinic-based care are now at higher risk of health deterioration [4,5]. These patients have been reported to face a higher risk of severe illness when infected with COVID-19. Thus, maintaining patient health status in the context of the current health care reconfigurations represents a significant challenge [5,6].

### Virtualizing Care During the COVID-19 Pandemic

To ensure access to care in light of restrictions to in-person visits, innovative virtual care models have been used during the pandemic as a necessary alternative [4,6]. The value of virtual care has been demonstrated in a number of studies through its ability to provide timely care that enables patients to maintain physical distancing while also conserving health care resources [6,7]. Among virtual care technologies, telemonitoring is being increasingly promoted as a tool to assist patients who need more frequent care touchpoints [7]. In the field of telehealth, telemonitoring has been defined as the use of technology to remotely monitor and transmit data relating to patient health status in geographically separated settings. With features enabling providers to closely monitor patient symptoms and vital parameters at home, the use of telemonitoring has been viewed as a valuable addition to the management of patients [8,9].

Since the pandemic started, large organizations such as the Canadian Cardiovascular Society and the Heart Failure Society of America have recommended transitions to virtual modes of care to meet the needs of patients during the pandemic [4,5]. In New York City, where high transmission of COVID-19 has been reported, telemonitoring was found to be an important component of the medical response to the pandemic because it reduced the demand on strained health care staff and enabled the meeting of patient needs at home [10]. As the health care system began to recognize the new reality and the presence of physical distancing restrictions, potential arose for virtual services such as telemonitoring to play an important role in patient management [3,10]. However, it is important to recognize that many telemonitoring programs were originally designed under the conditions of a pre–COVID-19 model of care that may no longer be available due to the limitations on in-person visits. With this growing shift to increasingly virtual health systems, there is a need to evaluate the functionality of a program under changing circumstances to optimize its use for conditions both during and beyond the pandemic.

The objectives of this paper were to (1) understand how telemonitoring is being used during the pandemic through the rapid expansion of a heart failure telemonitoring program, (2) report the barriers and facilitators related to the new virtual delivery of the telemonitoring program, and (3) evaluate the components of the telemonitoring program that require adaptation to sustain its use during and after the pandemic. In reflection of these objectives, the research question was: *With the changing conditions associated with the COVID-19 pandemic, what are the experiences and perceptions of patients, providers, and staff regarding the use of a telemonitoring program to support care needs?* 

## Methods

### Setting

**Figure 1:**
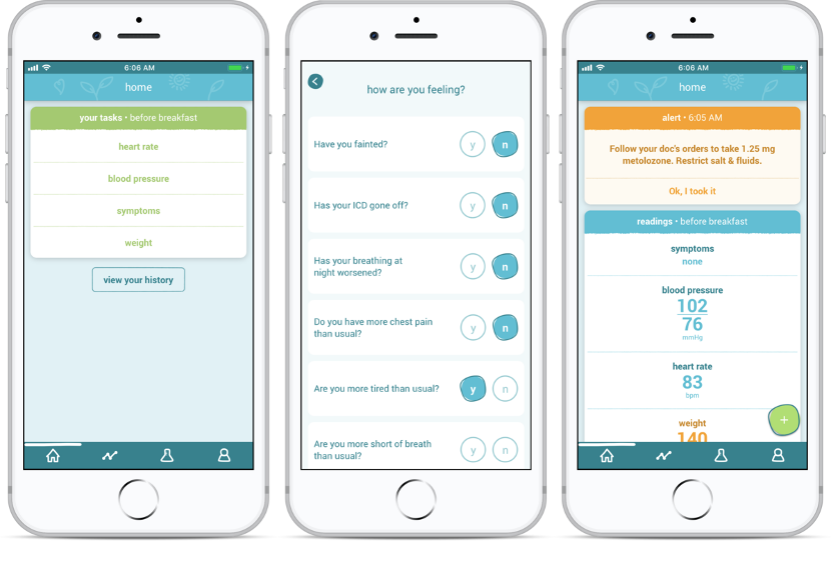
Screenshots of the Medly app displaying the patient self-care tasks, the symptom questionnaire, and the resultant self-care feedback after all tasks are completed and processed by the algorithm.

With the growing concern regarding COVID-19, the Peter Munk Cardiac Centre (PMCC) Heart Function Clinic at the Toronto General Hospital began to transition many of its services to a virtual care model. The majority of in-person appointments were rescheduled or replaced by videoconferencing visits or telephone calls. Clinicians affiliated with the PMCC Heart Function Clinic also had the option to enroll patients in the existing “Medly” program, a mobile phone–based telemonitoring program designed to provide remote clinical support for patients with heart failure [11,12]. The Medly program currently does not have any strict clinical criteria dictating patient eligibility for enrolment. Instead, cardiologists use their clinical judgement to refer patients they deem would benefit from the program. With Medly, patients use a smartphone with the Medly app, a weight scale, and a blood pressure monitor to record daily physiological readings and symptoms (Figure 1). Using these devices, patients are instructed to take daily weight and blood pressure readings as well as to record their symptoms through a series of self-report questions on the Medly app [12]. A rules-based algorithm uses these data to automatically generate individualized self-care instructions for the patients while simultaneously alerting clinicians when there are changes in the patient’s health status beyond their set clinical parameters [12]. Clinical alerts, delivered via email or the Web-based Medly dashboard, are mostly managed by the Medly Coordinator, a registered nurse within the Heart Function Clinic, with alerts being escalated to the cardiologists as needed. As the Medly system is a class II Health Canada–approved medical device, all data collected from the system are stored in the secure servers at the University Health Network, whereby patient information can only be accessed by the Medly Coordinator and the rest of the patient’s clinical team.

The Medly Coordinator also plays a fundamental role in the onboarding process, where during an in-person clinic visit, the treating cardiologist first presents the program to the patient and gains their approval for enrolment. The Medly Coordinator then meets with the patient to review the program processes (ie, account creation, training, study consent) and to assess their equipment needs [12]. Patients who require all equipment to be provided receive a full Medly kit, whereas other patients follow the bring your own device model and use some or all of their own devices (ie, smartphone, weight scale, blood pressure cuff) [12]. To support the rapid expansion of the Medly program during the COVID-19 pandemic, two nurses from the cardiology department within the Toronto General Hospital were seconded to assist with the foreseen increase in patient enrollment. As of March 8, 2020, the program had previously served 565 patients; however, in preparation for the uncertain impact of the pandemic and the need for remote support, plans were made to enable the Medly program to accommodate a rapid increase of up to 1000 patients. From March 9 to June 8, 2020, a total of 117 new patients were onboarded.

### Study Design

This study used a qualitative case study design [13] with the case defined as the telemonitoring program at the Heart Function Clinic at the Toronto General Hospital. The uses of an existing telemonitoring program by patients, clinicians, and operational staff were selected as the units of analysis to better understand the ways in which the pandemic has impacted their use and satisfaction of the Medly program.

### Recruitment of Participants

Patients were purposefully identified and recruited according to a range of demographic characteristics, including age, gender, location of residence (rural, suburban, urban), ethnicity, income, and comfort with technology. Although enrolment in the Medly program is at the discretion of each patient’s cardiologist, patients were considered eligible for this study if they spoke English and were users of the Medly program. We aimed to recruit patients who were onboarded to the Medly program both before and during the pandemic. To ensure that the patients onboarded during the pandemic had some experience using the system, only patients enrolled in the program for at least 20 days were considered to be eligible for the study. All 12 clinical staff members and 4 operational staff members of the telemonitoring program were invited to participate. All participants were recruited in alignment with an existing quality improvement study evaluating the telemonitoring program (University Health Network Research Ethics Board 16-5789 and University of Toronto Research Ethics Board 39449). Eligible patient participants were recruited until data saturation was reached, as in, no new perspectives or ideas were generated from the data. Specifically, as the data collection and data analysis processes were conducted on a continual basis, data saturation was deemed complete when no new insights were found from the latest 3 patients interviewed. This included, but was not limited to, patient feedback related to their experience using the program as well as challenges associated with the pandemic.

### Data Collection and Analysis

To accommodate physical distancing measures, in-depth semistructured interviews were conducted over the telephone by two authors experienced in qualitative research (AS and SW). Interview guides were developed based on the core components from the Benefits Evaluation Framework using semistructured, open-ended questions [14]. Each interview guide was developed and tailored for the patient, clinician (nurses and cardiologists), and operational staff participant groups. During each interview, participants were asked to comment on their experiences with managing heart failure both before and during the COVID-19 pandemic, as well as their experiences with the Medly telemonitoring program. Interviews were conducted between May 4 and June 18, 2020, and lasted approximately 30 minutes. All interviews were digitally recorded and professionally transcribed verbatim for analysis.

Interview transcripts underwent inductive thematic analysis at the semantic level according to the iterative 6-phase approach outlined by Braun and Clarke in 2006 [15]. Eakin and Gladstone’s “value-adding” approach to qualitative analysis was also used to enrich the analytic interpretation of the study findings and enhance the level of contextualization to the knowledge generated [16]. By using analytic devices such as reflexivity and generative coding to review each transcript, the authors were able to deepen their interpretation of the data in more abstract terms. Three authors were involved in the data analysis process (AS, MG, SW). Throughout the research process, all authors engaged in both procedural and analytical memoing to improve the overall trustworthiness of the analysis [17]. Transcripts and analytic memos were then entered into NVivo 9 (QSR International) to initiate the organization and analysis of the data. To gain a holistic perspective on all the data collected, one author (MG) independently analyzed all interview transcripts, whereas in parallel, two authors independently analyzed either the patient (AS) or clinician and staff (SW) transcripts. All authors subsequently met to discuss and compare codes for each participant group. Following the initial discussions, the codes were then grouped into categories to identify themes relating to the research question. After five analytic discussions, the research team collectively developed four themes. The four core themes were then individually reviewed by the study team to ensure internal coherence, consistency, and approval [15].

## Results

### Participant Characteristics

A total of 29 participants were interviewed: 16 patients, 9 clinicians (ie, cardiologist, registered nurse), and 4 operational staff. The characteristics of the interviewed patients are shown in Table 1. These data were collected via a self-report questionnaire as part of an existing quality improvement evaluation of the Medly program.

**Table 1:** Characteristics of the patient interview participants (N=16).

Characteristic		Value
Age (years), mean (SD; range)		54.5 (19.9; 23-78)
**Sex, n (%)**		
	Male	8 (50)
	Female	8 (50)
**Ethnicity, n (%)**		
	White	10 (63)
	Black	1 (6)
	Filipino	1 (6)
	South Asian	1 (6)
	Southeast Asian	2 (13)
	Not declared	1 (6)
**Place of birth, n (%)**		
	Canada	12 (75)
	Other	3 (19)
	Not declared	1 (6)
**Higher education achieved, n (%)**		
	High school	2 (13)
	Trade or technical training	4 (25)
	College or university	8 (50)
	Postgraduate	1 (6)
	Not declared	1 (6)
**Rurality, n (%)**		
	Urban	4 (25)
	Suburban	8 (50)
	Rural	3 (19)
	Not declared	1 (6)
**Living arrangement, n (%)**		
	Living with family/partner	13 (81)
	Living alone	2 (13)
	Not declared	1 (6)
**Income (CAD $),^a^** **n (%)**		
	<15,000	4 (25)
	15,000-49,999	3 (19)
	50,000-74,999	6 (38)
	>75,000	1 (6)
	Not declared	2 (13)
**Time of enrolment** **, n (%)**		
	Before March 2020	4 (25)
	After March 2020	12 (75)
**Participation model** **, n (%)**		
	Full kit	2 (13)
	Bring your own phone	4 (25)
	Bring your own everything	9 (56)
	Not declared	1 (6)
**Uses a smartphone** **, n (%)**		
	Yes	11 (69)
	No	3 (19)
	Not declared	2 (13)
**Frequency of smartphone use** **, n (%)**		
	Frequently	8 (50)
	Sometimes	5 (31)
	Not declared	3 (19)
**Comfort with technology** **, n (%)**		
	Very comfortable	3 (19)
	Somewhat comfortable	2 (13)
	Comfortable	4 (25)
	Not comfortable	2 (13)
	Not declared	5 (31)
**New York Heart Association classification** **, n (%)**		
	Class I	2 (13)
	Class II	9 (56)
	Class III	2 (13)
	Class IV	0 (0)
	Not declared	3 (19)

^a^CAD $1=US $0.79.

### Interview Findings

During the analysis of all participant data, four themes were identified: (1) providing care continuity through telemonitoring; (2) adapting telemonitoring operations for a more virtual health care system; (3) confronting virtual workflow challenges; and (4) fostering a meaningful patient-provider relationship.

#### Providing Care Continuity Through Telemonitoring

With the significant reduction of in-person clinic visits, the use of telemonitoring had become more desirable, as it provided clinically relevant patient data to help support virtual visits or the postponement of a visit. Although there are no strict clinical criteria guiding patient eligibility for Medly beyond requiring the patients to be followed by the Heart Function Clinic, clinicians increased the number of patients they referred to the program during the pandemic. With the limitations created by COVID-19, cardiologists were less selective in patient referrals to Medly, as their previous concerns for onboarding no longer weighed as heavily on their decision-making. Consequently, despite provider concerns, program nurses reported that patients with less familiarity with technology were able to use the application with minimal assistance:

One of the determining factors for me to onboard someone on Medly before COVID was if I had concerns about patients not being able to adhere to the daily weights reliably for whatever reason, I would hesitate to on-board them. Now that plays less of a role in my decision-making.

Cardiologist 3

Some of the patients are not always the most tech-savvy but they’re trying their best. And a few explanations on the phone and, you know, they’re good with it, you know?

Nurse 1

For many patients, with the heightened fear of contracting COVID-19 and the lack of accessibility to care services, Medly provided patients with a sense of security and comfort for their care management. New patients onboarded onto Medly appreciated the continual monitoring provided by the telemonitoring program, as it created a clinical safety net during a time when society was left in a state of uncertainty. The convenience and ease of being able to directly connect with a health care provider who was familiar with their medical history was also highlighted as a key value of the program:

It would be a wonderful thing for a lot of people in here to have somebody that had to check on them in the morning. I feel it's almost doing [it] to me because my vital signs don't change that much but at least it checks that I'm still alive and breathing.

Patient 2

My patients really liked the opportunity to be enrolled and be involved, they didn't know that it [Medly] existed before because they were sort of lower-risk patients who I hadn't thought to enroll in Medly before and now that they have some way to send in their vitals and have someone keep track of them a little more closely for a lot of patients, that gives them a lot of added security.

Cardiologist 1

Although clinicians were able to connect with patients through other modes of virtual engagement (ie, telephone calls, videoconferencing), they felt that Medly’s continual monitoring provided a more comprehensive picture of patient health compared to virtual visits alone. Specifically, clinicians using the Medly system were able to monitor trends in patient heart rate, blood pressure, weight, and symptoms, all of which helped detect worsening health and the potential need for patient follow-up. With the conditions of the pandemic and the absence of in-person visits, the metrics provided by Medly were highlighted to help provide a level of care continuity and context to support clinician decision-making:

I think it’s driving with your eyes open rather than closed and, to some degree, a lot of what you’re trying to do in terms of adjusting medications is to try and keep their weight within range and blood pressure and heart rate and their symptoms under control, and you can get all [of] that from Medly and track their trends over time.

Cardiologist 2

Beyond the usual benefits of telemonitoring, clinicians valued that Medly enabled them to make clinical assessments or changes in patient care that would usually be completed in-person. In the context of COVID-19 limitations, treatment optimization was still feasible, whereby clinicians were able to effectively titrate mediation without in-person patient visits to the hospital. Both patients and clinicians felt reassured that the quality of care had not been jeopardized under the shifting conditions of a virtual care model:

We’ve been able to titrate medication remotely, which is probably one of the biggest things because previously we would see patients in the clinic and then we wouldn’t change a new med until maybe the next time they were seen or a couple months down the road. I've been keeping track of many patients who are in the titration phase in their medication and we’ve been able to optimize their medications on a pretty rapid basis, like biweekly, to achieve triple therapy medication optimization, all while doing everything remotely.

Nurse 3

#### Adapting Telemonitoring Operations for a More Virtual Health Care System

With the rapid push for virtual care, many of the Medly program’s in-person processes were quickly virtualized to meet COVID-19 safety requirements. Despite common belief that the telemonitoring program was already functioning under a fully remote/virtual model, operational staff highlighted that while the onboarding process previously required patients to be introduced to the program following a clinic visit, these in-person procedures were now converted into a series of telephone calls with the Medly Coordinator ahead of a virtual visit. Clinicians and operational staff reported mixed patient reactions to the remote onboarding process; some reported that patients expressed confusion regarding why they were being enrolled in the program. Overall, the clinicians felt that patients had a more negative reaction in the early weeks of the pandemic, mainly because they had been preemptively contacted by the nurse coordinator for onboarding without receiving a contextual introduction from their cardiologist:

I think when patients have been seen in the clinic, in their virtual clinic, and then are enrolled onto Medly, their response is much better because they’ve already heard about it from their physician. Like, if they had had a clinic in the morning and then if you told them the nurse will call you today and get you set up, I want to remotely monitor you. That response has been really positive.

Nurse 3

One member of the operational staff reflected on the benefits of remote onboarding and found that the use of a low-contact setup enabled many patients to participate in the program without needing to leave their home. Specifically, with this new virtualized onboarding process, after the Medly Coordinator identified the type of Medly kit needed (eg, full kit, weight scale only), this equipment was delivered directly to the patient. However, clinicians and operational staff reported that this new process also resulted in delayed patient participation in the program, as some patients were unable to report their measurements due to their equipment still being in transit:

And then we’ve had to, like, mail out a lot of equipment to people who need it, and especially right now because people were not necessarily willing to go out and get their own equipment during COVID.

Staff 2

So, there’s a bit of like waiting involved whereas like normally you kind of expect the patients to be taking measures like the next day. In this case, it’s like potentially like one to two weeks later and then even then it’s kind of like, oh do you still not have your equipment yet or like how is that going?

Staff 1

As the pandemic progressed, clinicians and program staff reflected on the current operations of the Medly program and found that a number of patient tasks were more challenging within the context of COVID-19. For instance, many patients were required to obtain blood work for remote titrations; however, they felt that the risk associated with leaving their home outweighed the benefit of their treatment optimization. In addition, when patient symptoms worsened beyond their clinical parameters, clinicians or the automated Medly self-care feedback would direct patients to go to the hospital. However, this self-care advice was found to no longer be suitable, given the fear and heavy concern regarding virus exposure:

There are a number [of patients] that were quite concerned about going to get blood work, so they didn’t want to leave the house…It is a delicate balance on how much can we do through the phone, through Medly, make changes, but in the end we still need to get some of that data in order to make those clinical decisions.

Cardiologist 1

Patients have been also scared of going to hospitals. Even when we want them to come to the hospital to be assessed in person or to go to the emergency room many of them have pushed back and said that they’d rather deal with their symptoms at home rather than coming to the emergency room and expose themselves to a potential exposure to COVID-19.

Nurse 1

With physical distancing requirements in place, many program staff also noticed that patients residing at home were more often alone, without the presence of a caregiver. This loss of social support led many patients to experience a greater need for technical and emotional assistance that was normally provided by a visiting family member. With this, operational staff indicated that during the introductory period of the program, patients onboarded during the pandemic made more telephone calls requesting technical support than patients onboarded before the pandemic:

Some of the elderly patients have difficulty with technology where they haven’t been exposed to smartphones and that kind of thing. One of them actually said to me that he would usually get his kid to like show him how to do stuff or set stuff up on his phone, but because of COVID they haven’t been able to visit so he hasn’t been able to sort of get them to help.

Staff 1

#### Confronting Virtual Workflow Challenges

Clinicians experienced many challenges when using Medly in combination with other dedicated systems (eg, the electronic medical record, laboratory systems). Due to the lack of integration between the various systems, clinicians were required to navigate through multiple technologies to obtain up-to-date patient information. Clinicians felt that reviewing the patient information in a single system would not provide them with sufficient information to make a comprehensive clinic assessment. Thus, increased clinical workload was required to access multiple systems to fill the information gaps in their patient profiles:

When [radiology and cardiography] don’t appear it’s much harder to piece things together. So, when you’re seeing a patient, which is where it usually comes up, you want the data in one place. Like the last thing in the world I want to do is, I see a patient and I’m like “OK, I’m going to adjust the meds” –. But you [have] to go out of the EMR [electronic medical record]…then you have to log into the Medly platform which requires another password. You’re all doing this with the patient on the phone; it’s completely clunky.

Cardiologist 4

To accommodate the influx of patients during the rapid expansion, two nurses within the Toronto General hospital cardiology department were seconded to support the Medly program. The additional nurses enabled better balancing of the patient caseload. However, several issues regarding patient profile accessibility were reported. Specifically, the nurses indicated that it was difficult to access information about patients who were not part of their caseload. The nurses strongly desired a way to access information for patients not assigned to them to make it easier when they had to cover for one another:

*[One nurse] watched through all the alerts and so, with the ramping up of numbers more coordinators coming on, we had to find a way to change the caseloads and find who was watching [which patients]. So, there was a way where they changed it so we could easily identify which coordinator was the one watching which patient.*

Nurse 3

*[…] finding ways to look at different patients if they’re not your own. They’re separated* [now]*, but there* [should be] *a way to follow up with other patients if you were covering for another nurse and need to look up other patients.*

Nurse 4

Even with the increase in nursing staff, clinicians perceived an increase in their workload during the pandemic. Cardiologists were responsible for monitoring patient alerts over the weekends; however, responding to alerts became even more challenging with the increase in patient caseload. Ultimately, the clinicians thought that as more alerts would accumulate over the weekend, there could be a delay in the response time to contact the patients compared to during weekdays:

I have somewhere between 180 and 200 patients on Medly. So, on the weekend, without the nurse support, there’s a little bit more work for me to do.

Cardiologist 5

#### Fostering a Meaningful Patient-Provider Relationship

With the transition to a fully virtual model, patients onboarded during the COVID-19 pandemic felt that there was a lack of clarity regarding their purpose of enrollment into the program. Due to the restrictions associated with the pandemic, the Medly Coordinator was unable to provide patients with the initial face-to-face touchpoint that previous patients had received. Thus, many patients that were previously only seen every 6 months to a year were confused in regard to why they were required to input symptoms on a daily basis or form a relationship with the Medly Coordinator, who was previously not part of their care:

[Patients tell me]*, why do you suddenly want to hear from me every single day when prior to this you only saw me once a year… So, there are no negative responses; just a few people skeptical, “who are you and why are you calling me,” if they hadn't been pre-warned by their physician.*

Nurse 1

Clinicians also expressed that it was challenging to build a relationship with patients who were new to the Medly program. Multiple clinicians felt that they were not able to establish a personal connection or objectively assess their patients’ condition over the phone, as there was an absence of visual cues or caregiver support to obtain patient information. Clinicians indicated that patient body language often provided a good depiction of their well-being, and in cases where the patient was unable to vocalize their health issues, a caregiver would often assist in relaying relevant health information. Clinicians were concerned that without face-to-face visits or caregivers present, they would be unable to establish a relationship with patients or assess their condition accordingly:

What I have noticed is that, for new patients, it’s very hard to establish a good relationship... I called them a couple of times and we couldn't establish a kind of personal relationship. And I think that that was a bit of a problem. I think that that can increase their chances…of non-compliance.

Cardiologist 2

Regarding the frequency of interactions needed, clinicians had mixed views about how often they should be contacting patients over the telephone. One clinician found that a greater frequency of patient interactions strengthened the trust and basis of their patient relationship. However, other clinicians noted that most of their patient interactions were triggered by system alerts, and any further touchpoints with their patients would be burdensome to their workload:

Because the case load has increased, you do over time get a bit of an understanding for your … like any relationship, you need to kind of understand … what they prefer, what they are going through and how they would react.

Nurse 3

For patients, the perception of alert-driven relationships meant that they assumed clinicians would often assess patient well-being by the clinical parameters entered into the telemonitoring system. A lack of feedback from the nurses was usually perceived as a validation that the patient was doing well. However, patients who were not experiencing severe heart failure symptoms, and thus were not generating telemonitoring alerts, were dissatisfied because they were unable to establish a strong connection with their clinician. These individuals felt that many factors that contribute to their health were not being taken into consideration (eg, pain management, sleep, living conditions). With the influx of patients due to the pandemic, this issue around alert-driven relationships was further exacerbated, as clinicians faced an increase to their workload that made patient follow-ups beyond system alerts impractical:

If I’m the doctor and I'm sitting there, and I've got 100 people to talk to and I got a lot of things to do and all my numbers are perfect, [and I ask] “how are you feeling?” If [my doctor] was to ask me the question like [that] and I'm saying “well I'm not doing very well because I'm feeling like I've got severe pain and spasms in my hip, and my butt. And you're not addressing them.

Patient 3

## Discussion

### Principal Findings

Virtual care interventions have been implemented to provide solutions to the health delivery challenges posed by the COVID-19 pandemic [2,6]. Despite the push for virtual care and the previous support for its use, few studies have explored the perception of these technologies within the contexts of both before and during the pandemic [4]. In this study, we aimed to understand how changes to a heart failure telemonitoring program in response to the COVID-19 pandemic were perceived. From this study, we found that the expansion of the Medly telemonitoring program enabled clinicians to provide a level of cardiac care that would not be possible without telemonitoring. Our findings identified that to sustain the value of a telemonitoring program, it would be necessary to adapt its operational components according to the contextual circumstances (ie, required physical distancing due to the pandemic).

Prior to the COVID-19 pandemic, most health care encounters between patients and providers occurred face-to-face; thus, the incentive for virtual care was limited. The findings from this study indicate that by removing the safety net of in-person care, the restrictions of the pandemic acted as an enabler of virtual care adoption. We observed that many patients who would normally not be considered ideal candidates for the program were readily onboarded despite concerns regarding their technological or health literacy levels. Patients of varying backgrounds used the program beyond expected levels of success; this finding helped challenge the paradigm regarding the characteristics of an “ideal” program candidate [18]. Often, with these biases, many virtual care interventions fail to serve patient groups that are most in need of support [19]. Our findings suggest that re-evaluating the assumptions of who can benefit from a telemonitoring program may help expand the range of patient groups it serves.

While Medly was able to fill a void in patient care, it is important to recognize that the program team was in a unique position to adapt many of its underlying in-person operations. By virtualizing the onboarding process and the delivery of program equipment, the Medly team was able to accommodate the rapid expansion under the province’s physical distancing requirements. Other studies have reported that many heart failure clinics attempted to virtualize in-person practices but were unable to maintain these services due to the lack of infrastructure and clinical support available [4]. However, despite the Medly team’s success virtualizing the onboarding process, several systems (ie, Medly automated feedback) and clinician instructions were still deemed inappropriate (eg, laboratory work at a local clinic, advice to go to the hospital) in the context of the pandemic. Due to these challenges, similar studies have recommended the development of communication channels or regular consultations with patients to enable them to voice their concerns [20]. Providing patients with these opportunities would help to re-evaluate program components to meet their changing needs and would further empower them to manage their care.

Despite significant efforts to prepare for the rapid expansion, the lack of system integration and established collaborative processes led to escalation of the level of virtual workflow inefficiencies within the program [21]. While the additional nursing staff helped support the patient caseload, the telemonitoring program was operating under a fragmented ecosystem of virtual technologies (ie, the EMR, the Medly system, videoconferencing) that increased the complexity of clinical practices. With these technical challenges, recent research has recommended shifting traditional clinical workflows to a more digitally assisted pathway [6]. Specifically, to prevent duplicating tasks or spending excessive time navigating different systems, the adoption of dedicated communication channels and tailored workflows across multiple platforms would help to increase clinician collaboration and streamline data access.

While the rapid expansion was able to provide access to care and consistent remote management for a larger group of patients, the model of care did not change with disease severity. The clinical parameters were individualized to the patients; however, some perceived that the program was built on the principle of *problem-centered care* [22]. Many patients did not understand the purpose of their enrollment in the program, especially those who were not producing alerts, and they felt that this lack of clarity jeopardized their virtual relationship. Recent studies have attributed similar findings to the widening of the scope of telemonitoring programs to accommodate the limitations posed by the pandemic without considering the impact of the changes on clinical effectiveness or patient satisfaction [6]. We argue that to sustain telemonitoring adoption, clinicians should begin to clarify the specific objectives of the telemonitoring program to prevent possible misconceptions among patients. In addition, a more holistic assessment of each patient’s condition should be explored to enhance patients’ perceived value of the intervention. For instance, the investigation of psychosocial outcomes has been encouraged in the context of chronic illnesses [23], as monitoring a broader set of health indicators (eg, pain, sleep, mental health) has been reported to contribute to an objective assessment of a patient’s well-being [24].

### Recommendations

Patient self-management and remote monitoring tools represent an important avenue to deliver high-quality care in the community [25]. With no clear end in sight for the pandemic, health experts are now warning that physical distancing restrictions and other safety measures may be required for many more months or even years [6,26]. In light of our study findings, we offer four recommendations that can be used to help proactively adapt and scale up telemonitoring programs in the context of the increasingly virtualized health systems.

#### Revisit Scope and Eligibility for the TM Program 

Consult with clinicians, staff, and patients to evaluate the scope and expectations of the program under the conditions of the pandemic and beyond. Reflect on how this scope will impact or influence potential expansion efforts to scale the program to other health care settings (ie, disease of focus, intervention delivery). In line with this new scope, consider revisiting the patients served by the program in terms of age, ethnicity, gender, and geographical location to improve accessibility to more underserved groups [27].

#### Expand Information Modalities Available to Streamline the Remote Onboarding Process

Create an unsupervised (ie, self-paced) dynamic or static tool demonstration, such as an interactive in-app tutorial, to walk patients through key application features. These modalities should emphasize the purposes and patient-specific benefits of the program to foster a greater sense of connection with their care team (eg, recorded audio or video messages from clinicians, surveys to understand equipment needs and difficulties). Ensure that common troubleshooting scenarios are included in the demonstration (eg, expected response to an alert, how to manage a loss of connectivity) to help standardize the onboarding process for all patients.

#### Support Efficient Modes of Clinician Communication and Patient Management

Consult with clinicians to develop direct communication channels and pathways to streamline navigation across multiple virtual platforms (ie, synchronized and automatically updated EMR and telemonitoring dashboard data). To facilitate task-sharing, nurses should be given access to all patients’ profiles without shifting responsibilities for their assigned patient alerts.

#### Personalize Frequency and Patient Information Collected

Based on the evolving scope of the program, consider monitoring other health indicators (eg, pain, sleep, depression) to provide a more nuanced assessment of patient well-being in the absence of worsening heart failure symptoms. Consult with patients and clinicians to assess the frequency of touchpoints needed based on individual characteristics that may not be driven by alerts. Patient preferences regarding modes of communication, including video, text message, or audio, should also be incorporated to personalize care delivery.

### Limitations

There are a number of limitations to this study. First, among the group of clinicians who agreed to participate, 3 were unable to complete the interview due to scheduling challenges. Second, due to challenges posed by COVID-19, we used telephone-based recruitment and interview processes, which may have biased our patient sample toward individuals who are more comfortable with technology. Third, despite our intent to purposefully recruit patients across a range of demographic characteristics, patients in this study were mainly young, White, suburban, and/or college-educated. We recognize that the population of patients at the Heart Function Clinic is largely White and educated; therefore, our sample of patients for this study is reflective of the clinic population but may not be representative of the broader heart failure population in relation to age, ethnicity, rurality, and education. Finally, the recommendations from this study may not be reflective of all the requirements to adapt and scale telemonitoring programs, as it was based solely on the findings of one telemonitoring program for heart failure self-care. With this, we recognize that the recommendations outlined above may describe a subset of the key requirements to proactively adapt and scale telemonitoring programs under the context of increasingly virtualized health care systems due to the pandemic.

### Conclusions

The emergence of the COVID-19 pandemic has required society to re-evaluate virtual services as essential. The removal of traditional safety nets for in-person care has provided an opportunity for virtual services such as telemonitoring to demonstrate their value. In this study, we found that the rapid expansion of a telemonitoring program enabled patients to access cardiac care while maintaining physical distancing measures. Telemonitoring was able to fill a void for consistent patient monitoring, although the metrics provided by the system did not always represent an accurate picture of the patients’ well-being. Under the conditions of the pandemic, issues surrounding unintegrated, siloed systems (eg, EMR, laboratory systems) were escalated, as they contributed more to the clinician workload burden. To optimize the use of telemonitoring under the conditions of a “virtual world,” we present a series of recommendations to help sustain telemonitoring adoption through improvements in workflow efficiency and personalized care. These recommendations may not be inclusive of all the requirements to support the scalability of telemonitoring interventions; however, they will help serve as a foundational guide to better adapt program design. Ultimately, the intent of these recommendations is to improve care delivery for patients of varying needs and capabilities and to better support more patient-centered care both during and after the pandemic.

## Abbreviations

EMR: electronic medical record
PMCC: Peter Munk Cardiac Centre
